# Supplementary material for: Emissions and Exposures Associated with the Use of an Inconel Powder during Directed Energy Deposition Additive Manufacturing
Source: Int J Environ Res Public Health. 2023 Jun 22;20(13):6206. doi: 10.3390/ijerph20136206 (PMC10341570; doi:10.3390/ijerph20136206)
Supplement: Supplementary file 1 [file ijerph-20-06206-s001.zip › ijerph-2414067-supplementary.pdf]

International Journal of
Environmental Research and Public Health

MDPI

Supplementary Materials

Table S1. Particle shape analysis guideline.

Parameter	Short description	Result guide
Circularity	Proximity of a particle to a perfect circle.	1 = Perfect circle.<br>0 = Narrow or elongated particle.
Convexity	Surface smoothness or roughness of a particle.	1 = Smooth surface.<br>0 = Spiky or irregular surface.
Elongation	Width-to-length ratio.	1 = Elongated particle with increased width or length.<br>0 = Equilateral particle.

Table S2. Calculation of AER.

Repeat	Workshop	AM laboratory	Post-processing area	
			Natural ventilation	Extraction ventilation
1	22.4	25.5	18.8	18.1
2	20.5	23.8	11.3	42.4
3	26.2	26.4	21.7	32.2
4	-	20.9	-	-
Avg.	23.0	24.0	17.0	31.0

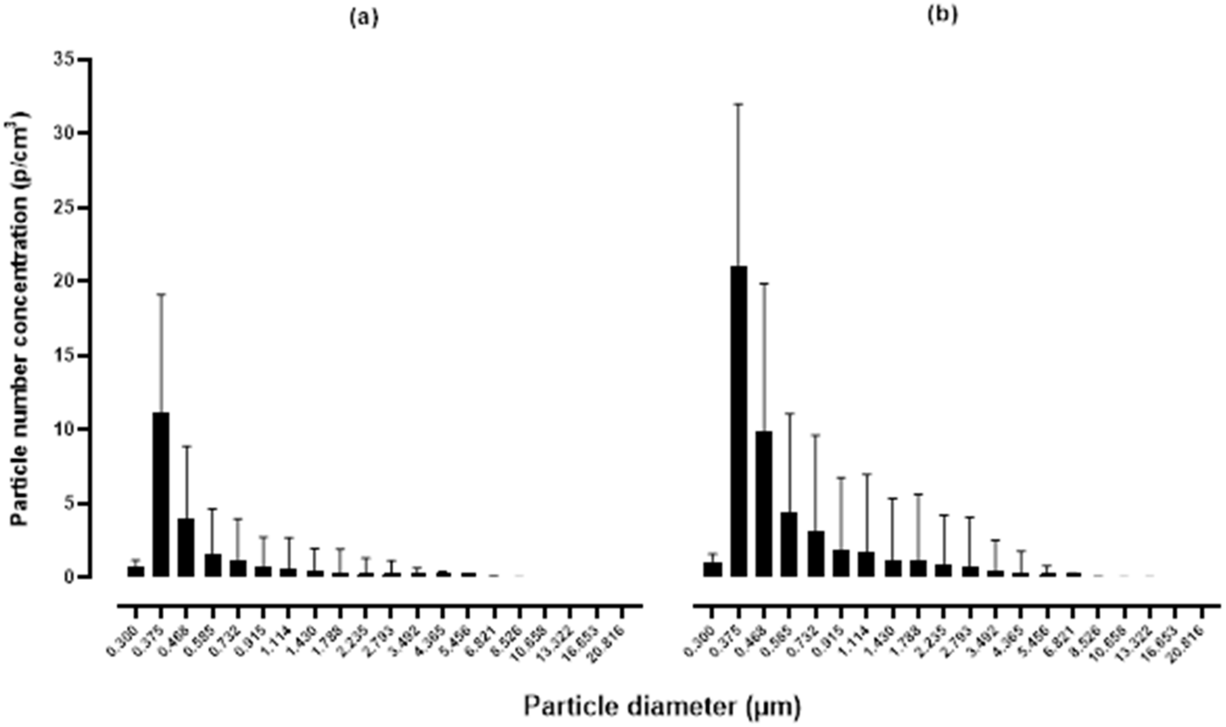

Figure S1. Particle size distribution chart displaying the most prominent size fraction during personal exposure throughout the AM process for (**a**) LENS and (**b**) LMD.
